# Neonatal seizures in a rural Kenyan District Hospital: aetiology, Incidence and outcome of hospitalization

**DOI:** 10.1186/1741-7015-8-16

**Published:** 2010-03-17

**Authors:** Michael Mwaniki, Ali Mathenge, Samson Gwer, Neema Mturi, Evasius Bauni, Charles RJC Newton, James Berkley, Richard Idro

**Affiliations:** 1 Centre for Geographic Medicine Research (Coast), Kenya Medical Research Institute, PO Box 230, Kilifi, Kenya; 2 Clinical Research Unit, London School of Hygiene and Tropical Medicine, London, UK; 3 Neurosciences Unit, UCL-Institute of Child Health, The Wolfson Centre, Mecklenburgh Square, London, WC1N 2AP, UK; 4 Centre for Clinical Vaccinology and Tropical Medicine University of Oxford Churchill Hospital Oxford, OX3 7LJ, UK; 5 Department of Paediatrics and Child Health, Mulago Hospital/Makerere University Medical School, Kampala, Uganda

## Abstract

**Background:**

Acute seizures are common among children admitted to hospitals in resource poor countries. However, there is little data on the burden, causes and outcome of neonatal seizures in sub-Saharan Africa. We determined the minimum incidence, aetiology and immediate outcome of seizures among neonates admitted to a rural district hospital in Kenya.

**Methods:**

From 1^st ^January 2003 to 31^st ^December 2007, we assessed for seizures all neonates (age 0-28 days) admitted to the Kilifi District Hospital, who were resident in a defined, regularly enumerated study area. The population denominator, the number of live births in the community on 1 July 2005 (the study midpoint) was modelled from the census data.

**Results:**

Seizures were reported in 142/1572 (9.0%) of neonatal admissions. The incidence was 39.5 [95% confidence interval (CI) 26.4-56.7] per 1000 live-births and incidence increased with birth weight. The main diagnoses in neonates with seizures were sepsis in 85 (60%), neonatal encephalopathy in 30 (21%) and meningitis in 21 (15%), but only neonatal encephalopathy and bacterial meningitis were independently associated with seizures. Neonates with seizures had a longer hospitalization [median period 7 days - interquartile range (IQR) 4 to10] -compared to 5 days [IQR 3 to 8] for those without seizures, *P *= 0.02). Overall, there was no difference in inpatient case fatality between neonates with and without seizures but, when this outcome was stratified by birth weight, it was significantly higher in neonates ≥ 2.5 kg compared to low birth weight neonates [odds ratio 1.59 (95%CI 1.02 to 2.46), *P *= 0.037]. Up to 13% of the surviving newborn with seizures had neurological abnormalities at discharge.

**Conclusion:**

There is a high incidence of neonatal seizures in this area of Kenya and the most important causes are neonatal encephalopathy and meningitis. The high incidence of neonatal seizures may be a reflection of the quality of the perinatal and postnatal care available to the neonates.

## Background

The first month of life is one of the highest risk periods for seizures [1] and seizures are the most common manifestation of neurological conditions in the neonate [2]. Neonatal seizures have been shown to be a major risk factor for inpatient death and subsequent neurological disability [3-5]. They show marked differences from seizures in older age groups: tonic-clonic seizures, common in children and adults, are rarely seen in the neonate [6] and electro-clinical dissociation [that is, seizure activity on electroencephalography (EEG) without clinically observed manifestations] are more common in neonates than in other age groups [7]. These differences can result in either over or under-estimation of the incidence of neonatal seizures [8]. In addition, the advancement in the treatment of neonatal seizures has changed little compared to seizures in the older population. Phenobarbital, introduced in 1914, remains the drug of choice especially in resource-poor settings [9,10].

Studies of neonatal seizures in developed nations show that most ictal events are secondary to acute neurological insults such as hypoxic ischaemic encephalopathy (HIE), stroke or infection [5,11]. In sub-Saharan Africa, HIE has also been strongly associated with many cases of early-onset neonatal seizures [12]. However, the presence of the identified risk factors for HIE, such as pH <7, Apgar score <5 at 5 min and the requirement for intubation, have proved to be unreliable predictors of neonatal seizures in this region [13]. In the short term, seizures in the neonate may be associated with higher inpatient death and prolonged inpatient stay [12,14]. In the long term, neonatal seizures are associated with an increased risk of neuro-disability including epilepsy, cerebral palsy and delayed neurodevelopment [4,15-17].

Although neonatal seizures are a common problem in hospitalized neonates in the region [12,18,19], few African studies have examined the burden and causes of seizures or the outcomes of treatment. The higher rates of neonatal encephalopathy, neonatal sepsis and premature births in developing countries [20] suggest that the incidence may be much higher than that reported in the West. In Ethiopia, one hospital based study found an incidence of 13.6 per 1000 live births [12]. More recently, in a study in which we examined the aetiology of acute seizures in Kenyan children, the incidence was found to be highest during the neonatal period [14]. In the current study, we recruited all neonates from the same defined geographical area of coastal Kenya hospitalized with seizures over a 5-year period in order to estimate the incidence, aetiology and outcome of hospitalization.

## Methods

### Location

Since 1998 we have conducted continuous inpatient surveillance aimed at describing the causes and features of common illnesses among Kenyan children in the Kilifi District Hospital [21,22]. The hospital is located within the Kilifi epidemiology and demographic surveillance system (EPI-DSS). This is a system where a resident population of over 240,000 living within a catchment area of 891 km^2 ^is continuously monitored and vital events updated every 4 months. The entire area was mapped using global positioning system and both mapping and population data are linked on-line to the hospital admission data.

Standardized clinical and laboratory data is prospectively collected on admission and at discharge or death by clinical and medical officers and directly entered into a computer database. For this analysis, we used data for patients admitted over a 5-year period from 1^st ^January 2003 through to 31^st ^December 2007, since this is the period during which we had reliable data. Consent for use of the data was obtained from the guardian of every individual child at point of admission and the study was approved by Kenyan National Scientific and Ethical Review Board.

### Study participants and definitions

For the purpose of this study, we defined a neonate as any child admitted aged 28 days or younger [23]. Seizures were defined as reported or observed repeated involuntary muscle contractions, abnormal tonic extensions or jerky movements of any part of the limb, face or mouth that was not stimulus sensitive or repetitive abnormal chewing, ocular or pedalling movements. The discharging clinician made the final diagnosis (for example, neonatal sepsis, neonatal encephalopathy or prematurity) after review of the admission history, inpatient management notes and laboratory investigations. These diagnoses were checked by a supervising clinician and followed the current World Health Organization (WHO) guidelines for the management of common illnesses in hospitals with limited resources [10]. The diagnoses are outlined in Appendix 1. A definite diagnosis of invasive bacterial disease was made upon isolation of pathogenic organisms from blood culture or cerebrospinal fluid (CSF). Otherwise sepsis was considered as the possible diagnosis in any newborn presenting with any one of the following signs: abnormal temperature (>37.5°C or <35.5°C) and multiple skin pustules, umbilical redness or pus, respiratory distress, lethargy, seizures or feeding problems [10]. Meningitis was defined as positive CSF culture or a white cell count of >50/μL in CSF or a positive bacterial antigen test or gram stain [24]. Prematurity was considered in any neonate born before 37 completed weeks if the last date of monthly period was known or in infants whose estimated gestation was less than 37 completed weeks [25]. Neonatal encephalopathy was considered in any newborn who had history of a poor Apgar score (<7) at 5 min (if delivered in hospital) with accompanying history and signs such as poor cry, feeding problems, restlessness, agitation, hypotonia, hypertonia, seizures and coma, or similar symptoms and signs after excluding other possible causes for the home-birth admissions. A diagnosis of neonatal jaundice was made when the total plasma bilirubin levels (as measured by the Neobil, Schuco International, London, UK, 2005) were elevated above an established threshold for phototherapy for the age, gestation, weight and clinical features [26]. We considered any neonate presenting with trismus or spasms occurring on stimulation or spontaneously with or without feeding difficulties to have neonatal tetanus. Since we recorded up to two final diagnoses recorded at discharge or death using all available clinical, laboratory and radiological information, the sum total of all final diagnoses may overlap or exceed 100%.

### Admission procedures

At admission, the clinician performed emergency care procedures such as correction of hypothermia, hypoxaemia, hypoglycemia and hypovolemia before taking a formal admission history. The history included the number and where possible a parental description of the seizure types and interventions already offered. The physical exam included a formal assessment of the gestational age [25], determination of a possible site of infection and any injuries. A venous blood sample was drawn (according to weight but not exceeding 3 mL) for a full blood count, blood glucose, electrolytes and microbiological culture. Hypokalaemia was defined as plasma K^+^<3.0 mmol/L, hyperkalaemia as K^+^>5.0 mmol/L, hyponatraemia (moderate-severe) as Na^+^<125 mmol/L and impaired renal function as plasma creatinine >80 μmol/L. Hypoglycaemia was considered in any child with a blood glucose <3 mmol/L. As part of the WHO guidelines, meningitis was considered in all neonates presenting with any one of: drowsiness, lethargy, reduced feeding, high pitched cry, irritability, apneic episodes, a bulging fontanel, unconsciousness or seizures and, where not contraindicated, a lumbar puncture was performed [10]. All received empirical antibiotic treatment (penicillin and gentamicin). A change of antibiotics and the duration of treatment were guided by the results of culture and the clinical response. There were no facilities to identify viral causes of meningo-encephalitis.

### Data management and statistical considerations

Admitting clinicians entered individual patient clinical data at point of contact using a FileMaker Pro database (5.5v1 Developer, FileMaker Inc, CA, USA). Likewise, laboratory data was double entered and verified using FoxPro for windows (FoxPro 2.5b for Windows, Microsoft Corporation, WA, USA). We used Stata 9.2 (Stata Corp, Texas, USA) for the final analysis. In order to calculate the incidence of neonatal seizures, the denominator, the population of live births (total and by gender) in the demographic surveillance area at the mid-point of the study (1^st ^July 2005) was estimated to be 708 of whom 339 were males. The estimate was based on a log-linear regression model of re-enumeration points dated between 27^th ^April 2001 and 30^th ^June 2007. The rates are expressed as events per 1000 live births per year. We then compared the clinical and laboratory features in newborns with and without seizures to describe the clinical risk and aetiological factors for neonatal seizures. We used Pearson's chi square test (or Fisher's exact test as appropriate) to compare proportions. We compared the medians of skewed data using the Mann Whitney-Wilcoxon's rank-sum test and applied the Mantel-Haenszel estimate to quantify the effect of seizures on inpatient death while controlling for weight. Data is presented with crude odds ratios (OR) and a *P*-value < 0.05 was considered significant. In order to determine the risk factors independently associated with seizures, we entered clinical and laboratory parameters with a *P*-value < 0.1 at univariable analysis into a multiple logistic regression model using a stepwise entry system.

## Results

### General description

**Figure 1:**
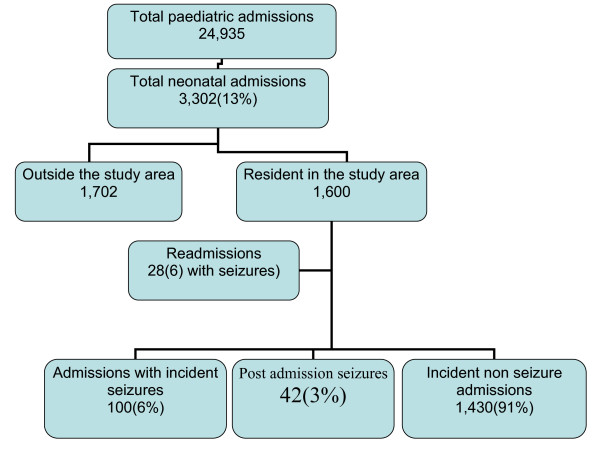
**Neonatal admission to Kenyan District Hospital from January 2003 to December 2007**.

From January 2003 to December 2007 a total of 24,935 children were admitted to the Kilifi District Hospital of whom 3302 (13.2%) were neonates (age ≤ 28 days, 60% males). Seizures were reported by parents or carers in 244 (7.4%) of all incident neonatal admissions irrespective of place of residence (study or non-study area). Of the 3302 neonates, we excluded 1702 (51%) admissions from outside of the study area (Figure 1).

### Incidence

Among the 1600 neonates identified from the study area, 28 (1.8%) were readmissions and six of these had seizures. We excluded these non-incident admissions from further analysis. One hundred (6%) neonates out of the 1572 incident admissions from the study area were admitted with seizures. In addition, another 42 neonates developed seizures after admission to the ward. Thus, there were a total of 142 (9%) neonates with seizures among the incident admissions from the study area. Overall, the incidence of neonatal seizures was [39.5 ([95% confidence interval - CI - 26.4-56.7)] per 1000 live births per year. The incidence was [13.6 (95% CI 9.6-17.2)] per 1000 live births in the first 48 hrs, [8.5 (95% CI 6.1-12.3)] per 1000 live births in the next 2 to 7 days of life and [18.6 (95% CI 15.4-23.0)] in the rest of the neonatal period. The incidence did not differ by sex (χ^2 ^= 0.005, *P *= 0.95).

Two thirds of all neonatal admissions were delivered at home (out-born) of whom 100/1,047(9.6%) had seizures compared to 42/525 (8.0%) born in hospital (χ^2 ^= 1.02, p = 0.31). The prevalence of seizures increased with birth weight. Seizures were reported in 3/196(1.5%) of neonates with a birth weight <1.5 kg, in 7/159(4.4%) of those with weights 1.5-<2.0 kg, 27/238(11%) of those with weights 2.00-2.49 kg and 105/967(11%) of those with a birth weight ≥ 2.5 kg, (χ^2 ^for trend = 4.92, P < 0.001).

### Clinical presentation, laboratory findings and diagnosis in neonates with seizures

#### Clinical presentation

Forty six of the 142 neonates (32.3%) had seizures within the first 48 hrs of life, 30 (21.1%) had seizures on days 3-7 and in the remaining 66 (46.5%), seizures occurred after the first week of life. Over 50% of seizures occurred within the first week of life and the majority of neonates, 79/142 (56%), had more than one episode within 24 hrs. The median number of seizures on presentation was three [interquartile range [IQR] 2 to 7). However, there was no significant difference between the number of seizures among newborns who died and those discharged alive, (Wilcoxon rank-sum test, *P *= 0.34).

Neonates with seizures were older at presentation compared to those without seizures and the median age of neonates with seizures was 6 (IQR 2 to 15) days compared to 4 (IQR 0 to 11) days for those without seizures, *P *< 0.001. Although a history of fever was more common in newborns presenting with seizures, there was no difference in the proportion of seizures among those with and without an axillary temperature of ≥ 37.5°C. The duration of fever in the two groups was also similar (Table 1).

**Table 1:** Clinical and laboratory features at admission.

Characteristics	Neonates with seizures, *n *= 142	Neonates without seizures, *n *= 1430	Crude odds ratio [OR (95 CI)]	*P *value	Adjusted OR (95% CI)	*P *Value
Median (interquartile range) age, days	6 (2-15)	4 (0-11)	1.04 (1.01-1.05)	0.002	1.01 (0.99-1.03)	0.44

Males (%)	83 (58.5)	840 (58.7)	1.12 (0.75-1.49)	0.75	-	-

Median weight (kg) [95%CI]	2.94 [2.46-3.29]	2.74 [2.04-3.18]	6.21(1.0-38.7)	0.05	1.00(0.99-1.00)	0.52

Fever (%)	75 (52.8)	502 (35.1)	2.14 (1.52-3.03)	<0.001	1.63 (1.10-2.43)	0.02

Irritability (%)	11(7.7)	25 (1.7)	4.61 (2.22-9.57)	<0.001	2.53 (1.04-6.17)	0.04

Inability to breast feed (%)	36 (25.4)	503 (35.2)	1.58 (1.08-2.33)	0.02	1.13 (0.71-1.81)	0.53

Bulging fontanel (%)	14 (9.9)	16 (1.0)	10.22 (4.9-21.1)	<0.001	5.94 (2.65-13.30)	<0.001

Hypoglycaemia (%)	23 (16.2)	254 (17.7)	0.91 (0.58-1.44)	0.69	-	-

Hyponatraemia (%)	4 (2.8)	43 (2.9)	0.92 (0.32-1.59)	0.87	-	-

Hypernatraemia (%)	3 (2.1)	9 (0.6)	3.34 (0.89-12.47)	0.07	-	-

Hypokalaemia (%)	12 (8.1)	130 (8.9)	0.90 (0.49-1.67)	0.75	-	-

Hyperkalaemia (%)	14 (9.5)	119 (8.2)	1.18 (0.66-2.11)	0.58	-	-

Positive cerebral spinal fluid culture (%)	9 (8.4)	11 (1.9)	14.01 (6.01-32.47)	<0.001	14.08 (3.60-55.09)	<0.001

Positive blood culture (%)	10 (7.0)	76 (5.3)	1.32(0.67-2.61)	0.80	-	-

### Laboratory findings

The blood glucose concentration at admission was documented in 1227 (78%) of the resident and incident neonatal admissions. Hypoglycaemia was observed in 277/1227 (22.3%) neonates. The proportions with hypoglycaemia at admission were similar in those with [23/125 (18%)] and without seizures [254/1,102 (23%)], χ^2 ^= 1.4, *P *= 0.24. The proportions of other key laboratory and clinical characteristics of those with and without seizures are as shown in (Table 1).

Of the clinical and laboratory features (fever, irritability, age, inability to breast feed, bulging fontanel and isolation of a pathogenic organism in CSF) that were associated with seizures on univariable analyses, only fever, irritability, bulging fontanel and isolation of a pathogenic organism in CSF were independently associated with seizures (Table 1).

### Diagnoses

The most frequent discharge diagnosis among neonates with seizures was neonatal sepsis 85 (60%), followed by neonatal encephalopathy 30 (21%) and neonatal meningitis 21 (15%) but only a diagnosis of neonatal encephalopathy or neonatal meningitis were independently associated with seizures (Table 2). Lumbar puncture (LP) for the evaluation of meningitis was more likely to be performed in neonates presenting with seizures; 107/142 (75%) of neonates with seizures had a (LP) compared to 569/1430 (40%) of those without seizures, χ^2 ^= 67, *P *< 0.001. In addition, among neonates that had an LP, an infectious agent was more likely to be isolated in CSF if the neonate presented with seizures [9/107 (8.4%) compared to 11/569 (1.9%)] of those without seizures, χ^2 ^= 31.4, *P *< 0.001.

**Table 2:** Clinical diagnosis and neonatal seizures.

Characteristics	Incident neonatal admissions with seizures, *n *= 142 (%)	Incident neonatal admissions without seizures, *n *= 1430 (%)	Crude odds ratio (95% confidence interval)	*P *value
Neonatal sepsis	89 (62.7)	772 (54.0)	1.27 (0.91-1.80)	0.16

Prematurity	3 (2.1)	248 (17.3)	0.10 (0.03-032)	<0.001

Neonatal encephalopathy	30 (21.1)	197 (13.7)	1.63 (1.06-2.50)	0.03

Neonatal jaundice	17(12.0)	372 (26.0)	0.38 (0.23-0.64)	<0.001

Meningitis	21(14.8)	8 (0.6)	34.4 (14.65-76.26)	<0.001

Neonatal tetanus	3 (2.1)	18 (1.3)	1.04 (0.48-5.70)	0.42

Although sepsis or meningitis was frequently suspected in neonates with seizures, an infective agent in either blood or CSF was isolated in only 16 of the 142 (11.3%) neonates with seizures. Nine out of sixteen of these had isolates grown from CSF. A diagnosis of bacterial meningitis was made in 21 (15%) of the cases with seizures. In nine cases, this was from a positive CSF culture. The commonest isolates from CSF were *Streptococcus pneumoniae *(three cases) and non-typhoidal salmonella (two cases). There was one case each of beta haemolytic streptococci, *S. aureus*, *Escherichia coli *and *Pseudomonas aeroginosa*. The diagnosis of meningitis in the other 12 neonates was based on an elevated CSF white cell count ≥ 50 μL.

A diagnosis of neonatal encephalopathy was made in 227/1572 (14%) of all incident neonatal admissions from the study area. A higher proportions [30/227(13.2%)] of the cases of neonatal encephalopathy had seizures compared to all other diagnosis, [112/1345(8.3%)], χ^2 ^= 5.7, *P *= 0.02.

Neonatal encephalopathy was the leading diagnosis in neonates admitted with seizures within the first 48 h of life, 28/46 (60.1%). Only two of this had meningitis and the remaining 16 had neonatal sepsis, three of whom had culture positive sepsis. After the first 48 h, but within the first week of life, neonatal sepsis was the predominant diagnosis. However, meningitis was diagnosed in six cases. Meningitis was also common after the first week of life (13/66 [19.7%]) although neonatal sepsis (43/66 [65.2%]) was still the commonest diagnosis.

### Immediate outcome of neonatal seizures

Neonates with seizures had a longer hospitalization. The median period of inpatient stay among neonates with seizures was seven (IQR 4 to 10) days compared to five (IQR 3 to 8) for those without seizures, *P *= 0.02.

Three hundred of the 1572 (19.1%) neonates died. Neonates with low admission weight (<2.5 kg) had a higher case fatality 194/593 (33%), compared to those weighing (≥ 2.5 kg) 97/967 (10%), χ^2 ^= 125, *P *< 0.001. There was no difference in case fatality between neonates with and without seizures, χ^2 ^= 0.76, *P *= 0.38. However, if this was stratified by weight, seizures were associated with higher case fatality in neonates ≥ 2.5 kg compared to low birth weight infants [<2.5 kg (OR 1.59 [95% CI 1.02 to 2.46), *P *= 0.037].

Of the 32 deaths in neonates with seizures, 12 (38%) were suspected to have sepsis and five (16%), meningitis. However, an infective organism was isolated in only four of these. Of the remaining babies, eight (25%) had neonatal encephalopathy. four had severe neonatal jaundice and three had tetanus. In addition, seven (22%) children who died were hypoglycaemic on admission and 14 (44%) had an abnormal core temperature (<36.0°C or >37.5°C). In total 15 (47%), of the neonates with seizures who died were low birth weight (<2.5 kg) with three (10%) weighing <1.5 kg.

On discharge, 14/110 (13%) of the surviving neonates had an abnormal neurological exam. Of the 14, four had hydrocephalus, three were discharged on long-term phenobarbital due to recurrent seizures, another three had a poor sucking reflex and two had spasticity. The remaining two newborns though not classified as normal had subtle undefined neurological abnormality. Follow up through our surveillance system showed that 16/1271 (9.1%) of all neonates died post-discharge and there was no difference between those admitted with or without seizures χ^2 ^= 1.78, *P *= 0.18.

## Discussion

Children living in this rural area of Kenya experience a very high burden of acute seizure disorders. In a previous report, we indicated that the minimum incidence of acute seizures in children 0-13 years of age is as high as 425 per 100,000/year [14]. Although available data suggests that children younger than 5 years have an even higher burden of acute seizure disorders and that neonatal diseases are major aetiological factors [4,14,27], the burden and causes of acute neonatal seizure disorders in rural African settings has been poorly documented [12,14]. In this study, the incidence of neonatal seizures was 39.5 (95% CI 26.4-56.7) per 1000 live births and the most common diagnoses associated with seizures were neonatal encephalopathy and meningitis.

### The incidence of acute neonatal seizures

The incidence data we describe is the minimum since not all neonates would have attended the Kilifi District Hospital. In the earlier study, it was estimated that only 20% of children with seizures in the community may have been admitted to hospital [14]. It is also worth noting that up to two-thirds of deaths in children <5 years in this community occur outside the hospital and this proportion may even be higher among neonates since the majority are born at home [20,28]. It is plausible that many neonates with seizures may be dying in the community before reaching the hospital. Moreover, distance from hospital also affects hospital attendance [14]. Thus, our data could have greatly underestimated the incidence of seizures in neonates in this community. Despite the above caveats, the study suggests that the incidence of neonatal seizures in this setting is much higher than that reported from developed nations [4,27] and this may reflect the high prevalence of risk factors for neonatal brain insult in this community.

Very few other centres in developing countries have attempted to quantify the burden of neonatal seizures. Previously, based on a small sample of just 43 neonates, we estimated the incidence to be 14.0 per 1000 live births [14]. Similarly In Ethiopia, the incidence was found to be 13.6 per 1000 live births [12] but the report only included early onset seizures (those occurring within 48 hrs of life) which may explain the much lower incidence. This study, with a longer surveillance period (5 years) covering the entire neonatal period, may present a more accurate estimate of the overall burden of neonatal seizures in the region.

### Diagnoses, inpatient outcome and implications for public health

Although neonatal sepsis was a common diagnosis, it was not independently associated with seizures. Importantly, isolation of a pathogenic organism in blood alone was not associated with seizures. The two principal diagnoses associated with seizures were neonatal encephalopathy and meningitis. The increased inpatient case fatality associated with seizures supports previous reports [3,5]. However, it is worth noting that this difference was only significant for the term-weight newborn. The lack of association between seizures and death in the low-weight neonates may have been veiled by the very high case fatality (>30%), especially in the preterm babies.

Although debate continues on whether seizures in the neonatal period cause brain damage or are just an epiphenomenon of insults already sustained from the underlying pathology, available evidence suggest that long-term outcome is largely dependent on the cause of the seizure [3,5]. Our findings, that up to 13% of all newborn with seizures had neurological abnormalities at discharge, is supported by community-based studies in this region that identified neonatal insults as the key risk factor for moderate and severe neurological impairment [29]. However, higher levels of neurological abnormalities at discharge and on long-term follow up have been reported in studies in developed countries [3-5]. It is plausible that the relatively lower prevalence of neurological abnormalities on discharge may be a reflection of the very high inpatient neonatal death. Likewise, the high post discharge neonatal deaths (close to 10%), irrespective of seizures, may have an implication on prevalence of neurological abnormalities in the long term.

The two most common diagnoses associated with neonatal seizures were infections and neonatal encephalopathy. Both conditions are preventable and, using simple public health interventions, it should be possible to reduce the burden of neonatal seizures and neonatal brain injury in the region. Broad measures, such as ensuring safe deliveries, appropriate neonatal resuscitation and prevention and early treatment of infections in the newborn period, may reduce this burden [30].

In this study, we were only able to document seizures that were clinically evident. It is possible that we missed subclinical seizures and, given the fact that electro-clinical dissociation is more common in neonates than in other age groups[7], the electroencephalograph monitoring could have led to our further underestimating the burden of seizures. Secondly, for out-born neonates, the Apgar scores were not available and so we used the history and the physical state of the child on admission to make the diagnosis of neonatal encephalopathy. Thirdly, neuro-imaging was unavailable and so we are unable to establish other causes of seizures such intracranial and periventicular haemorrhages. Fourth, as many of the births occurred at home and there was a lack of any consistent data on gestational age, we were unable to examine the effect of gestational age on the prevalence of seizures and on outcome. Finally, this study did not examine in detail the long-term outcome of neonates with seizures.

## Conclusion

In conclusion, newborns in this rural area of Kenya have a high incidence of seizures and neonates with seizures have a longer hospitalization, increased case fatality in heavier neonates and neurological deficits on discharge. The main causes of seizures are birth-related complications such as neonatal encephalopathy and infections, especially meningitis. Public health interventions aimed at ensuring safe deliveries, appropriate neonatal resuscitation and prevention of infections in the newborn period may help to reduce the high incidence. Prospective studies on the long-term neurological and developmental outcome following neonatal seizures are needed.

## Abbreviations

CSF: cerebral spinal fluid; CI: confidence interval; EPI-DSS: epidemiology and demographic surveillance system; HIE: hypoxic ischaemic encephalopathy; IQR: interquartile range; LP: lumber puncture; OR: odds ratio; WHO: World Health Organization.

## Competing interests

The authors declare that they have no competing interests. The Wellcome Trust supported this study but played no role in the design, conduct of the study, the analysis and interpretation of data or in the preparation, review and approval of the manuscript.

## Authors' contributions

MM designed the study, collected and analysed the data and wrote the draft. AM and SG provided inpatient care and assisted in data analysis. NM and JB supervised clinical care and were involved in writing the draft. CN suggested the study, participated in study design and was involved in data analysis. EB provided the mid-study population data and information on the EPI-DSS. RI designed the study, participated in data collection, analysis and interpretation. All authors critically reviewed the manuscript.

## Appendix

### Definitions of discharge diagnoses

***Invasive bacterial disease: ***Positive blood or CSF cultures.

***Possible sepsis: ***Presentation with either one of the following:

• abnormal temperature (>37.5°C or <35.5°C),

• multiple skin pustules,

• umbilical redness or pus,

• respiratory distress, lethargy, seizures or feeding problems.

***Meningiti: ***Presentation with either one of the following:

• positive CSF culture,

• white cell count of >50/μL in CSF,

• positive bacterial antigen test or gram stain.

***Prematurity: ***Documentation of gestation age below 37 completed weeks by:

• where last monthly period is known and expected date of delivery can be calculated,

• acceptable gestation age estimation criteria with:

◦ ultrasound,

◦ the Dubowitz.

### Neonatal encephalopathy

• No cry at birth, poor Apgar score (<7) at 5 min.

• An accompanying history of feeding problems, restlessness, agitation, hypotonia, hypertonia, seizures and coma, or similar symptoms and signs after excluding other possible.

***Neonatal jaundice: ***Significant total plasma bilirubin levels for age, weight and clinical picture of newborn requiring at least phototherapy.

*Tetanus:*

• spasms that may be provoked or spontaneous,

• trismus.

## Pre-publication history

The pre-publication history for this paper can be accessed here:

http://www.biomedcentral.com/1741-7015/8/16/prepub
